# Identification of VGF nerve growth factor inducible-producing cells in human spinal cords and expression change in patients with amyotrophic lateral sclerosis

**DOI:** 10.7150/ijms.39101

**Published:** 2020-02-04

**Authors:** Yasuhiro Noda, Miruto Tanaka, Shinsuke Nakamura, Junko Ito, Akiyoshi Kakita, Hideaki Hara, Masamitsu Shimazawa

**Affiliations:** 1 Molecular Pharmacology, Department of Biofunctional Evaluation, Gifu Pharmaceutical University, 1-25-4 Daigaku-nishi, Gifu 501-1196, Japan.; 2 Department of Pathology, Brain Research Institute, Niigata University, Niigata, Japan.

**Keywords:** amyotrophic lateral sclerosis, VGF nerve growth factor inducible, motor neuron

## Abstract

Amyotrophic lateral sclerosis (ALS) is a serious disease characterized by the degeneration of motor neurons resulting in muscle weakness and paralysis. The neuroendocrine polypeptide VGF is localized in the central nervous system and peripheral endocrine neurons and is cleaved into several polypeptides with multiple functions. Previous studies revealed that VGF was decreased in the cerebrospinal fluid of ALS model mice and sporadic ALS patients. However, it is unknown which cells supply VGF in the spinal cord and a detailed localization is lacking. In this study, we evaluated the VGF-producing cells and protein localization using *in situ* hybridization and immunostaining in the spinal cords of ALS and control patients. *VGF* mRNA was localized both in the dorsal and anterior horns of the spinal cords. Moreover, in the anterior horn, *VGF* mRNA co-localized with a neurofilament heavy chain, which is a motor neuron marker, and *VGF* mRNA-positive motor neurons were decreased in the spinal cords of ALS patients. We revealed that VGF protein level was decreased in the anterior horn of ALS patients; however, the expression level of VGF protein was not changed in the posterior horn or white matter. Furthermore, the expression level of VGF protein was conserved in ALS patients with long-term survival. These results reveal that VGF is mainly supplied by human motor neurons, and suggest that VGF expression changes may be involved in ALS pathology.

## Introduction

Amyotrophic lateral sclerosis (ALS) is a severe neurodegenerative disorder causing the loss of motor neurons in the spinal cord, motor cortex, and brainstem. ALS is characterized by muscular atrophy resulting in dysarthria, dysphagia, respiratory problems, and fatality within 3-5 years 1. Whereas the cause is unknown in 90-95% of ALS cases, the remaining 5-10% of patients have a genetic background, such as a mutation of copper-zinc superoxide dismutase 1 (SOD1), C9orf72, or TAR DNA Binding Protein (TARDBP) 2. However, there are no effective therapeutic options or biomarkers, which are useful for differential diagnosis in early stages.

VGF (non-acronymic) was discovered as a nerve growth factor inducible protein in the rat pheochromocytoma PC12 cell line 3. VGF is a secreted polypeptide and processed by prohormoneconvertases 1 (also known as PC3) and 2 4 resulting in the production of various bioactive peptides, such as TLQP-62 (VGF_556-617_), TLQP-21 (VGF_556-576_), HHPD-41 (VGF_578-617_), AQEE-30 (VGF_588-617_), and LQEQ-19 (VGF_599-617_), and neuroendocrine regulatory peptides-1 (VGF_281-306_) and -2 (VGF_310-347_). These peptides are localized in neuronal and neuroendocrine cells in a tissue-specific manner 5-7 and exert specific neuronal bioactivities including an antidepressant effect, neurogenesis, and regulating energy expenditure 8-11.

Previous studies have suggested that VGF expression is decreased in the spinal cord, cerebral spinal fluid, and plasma in sporadic ALS patients and ALS model mice 12-15. Furthermore, there are several reports of the neuroprotective effects of VGF; 1) suppressing the death of cultured retinal ganglion cells 16, 2) enhancing the survival of cortical neurons 7, 3) VGF-derived peptide, AQEE-30, suppressed cell death in a model of Huntington's disease 17, and 4) induction of VGF by a pharmacological agent prolonged survival in a mouse model of ALS. These findings suggest that VGF and its derived peptide may be modulators of ALS progression. Although VGF C-terminus peptides were found in motor neurons of mice, it is still unknown which cell types supply VGF and whether these cells are responsible for disease progression in ALS patients.

In this study, we investigated the *VGF* mRNA distribution in human spinal cord by *in situ* hybridization because VGF is a secreted protein. Subsequently, *in situ* hybridization and immunostaining performed simultaneously in *VGF* mRNA located area to investigate the cell types which produce VGF and expression change of VGF in ALS patients. Lastly, we confirmed localization of VGF protein in human spinal cord.

## Method

### Ethical considerations

Clinical procedures were performed in accordance with the latest version of the Helsinki Declaration (http://www.wma.net/en/30publications/10policies/b3/). Written informed consent for autopsy, collection of samples and subsequent use for research purposes was obtained from the next of kin of the deceased involved in the study. This study was approved by the Ethics Committees of both Gifu Pharmaceutical University (Approval number; 30-31) and Niigata University School of Medicine (Approval number; 2523).

### Spinal cords of ALS and control patients

Spinal cords were taken at autopsy from nine patients with sporadic ALS (age 70.9 ± 2.7 years), who were either bulbar or upper-limb onset, and otherwise taken from 3 patients with ALS, who showed unusually long-term survival 18. Spinal cords from nine individuals, without a history or evidence of neurological disease, were also taken at autopsy as a control (age 69.8 ± 4.6 years). The clinical data for these 21 patients are summarized in the Table 1. The spinal cord was immersed in 20% buffered formalin (pH 7.6) for approximately 4 weeks. Multiple transversely cut tissue-blocks of the cervical and lumbar cords were embedded in paraffin-wax and maintained in the Brain Research Institute of Niigata University until slicing at room temperature. Samples were cut at 4 µm thickness and attached to MAS-coated glass (Matsunami Glass Ind. Ltd.). Paraffin-embedded transverse sections of the lumbar and cervical cords were made at Niigata University, and the paraffin sections were transported to Gifu Pharmaceutical University for the immunohistochemical and *in situ* hybridization study.

### *In situ* hybridization

*In situ* hybridization was performed using RNAscope^®^ 2.5 HD Detection Kit (Bio-techne, MN, USA) following the manufacturer's instructions. After baking the slides in a dry oven (1 h at 60°C), the slides were immersed in xylene (2 times 5 min), 100% EtOH (2 times 1 min), and air-dried for 5 min for deparaffinization. Hydrogen peroxide was added to each section for 10 min at RT and washed the slides in distilled water (2 times). The slides were submerged in boiling 1X Target Retrieval solution for 15 min and then immediately immersed the slides in distilled water (2 times) and 100% EtOH. After air-drying, Protease Plus solution was added to each section, incubated (40°C for 30 min), and washed slides in distilled water (2 times). Hybridization was performed using a human VGF Probe (RNAscope^®^ Probe-Hs-VGF; Cat # 466111, Bio-techne) or negative control siRNA (RNAscope^®^ Negative Control Probe-DapB; Cat #310043, Bio-techne). The probes were dropped on each section and incubated the sections in humid conditions (2 h at 40°C). After incubation, the slides were washed in 1X Wash Buffer (2 min in RT 2 times). Subsequently, following reactions were performed, Amp 1 (30 min at 40°C), Amp 2 (15 min at 40°C), Amp 3 (30 min at 40°C), Amp 4 (15 min at 40°C), Amp 5 (30 min at RT), and Amp 6 (15 min at RT). After each incubation, the slides were washed with 1X Wash Buffer (2 min at RT 2 times). RED solution (RED-A:RED-B = 60:1) was dropped onto each tissue section (10 min at RT), the slides were washed 3-5 times in distilled water, and then incubated with DAPI (1:1,000, 15-25°C, 30 min; Biotium) for nuclear staining. After washing with phosphate-buffered saline (PBS), sections were encapsulated using fluoromount (Diagnostic BioSystems). For the experiments in figures, samples after treatment with RED solution were used for immunostaining.

### Immunohistochemistry

The paraffin sections were immersed in xylene (15 min, 3times), anhydrous ethanol (10 sec, 2 times), 99% ethanol (10 sec, 2 times), 90% ethanol (10 sec), 70% ethanol (10 sec), and distilled water (10 sec) for deparaffinization. Sections were activated by immersion in citrate buffer (pH 6.2, 120°C, 15 min). Each section was washed with PBS and incubated with 10% horse serum for blocking (15-25°C, 1 h; Vector Laboratories, CA, USA).

In the experiment with double immunostaining (Fig. 5), each section was washed with PBS, and incubated with mouse anti-neurofilament H non-phosphorylated (NFH) monoclonal antibody (SMI-32) (1:1000; Cat #NE1023, Lot D00168792, Merck Millipore Corporation, MA, USA) and rabbit anti-VGF polyclonal antibody (1:500; Cat ab69989, Lot GR130693-3, Abcam, Cambridge, UK). After washing with PBS, sections were incubated with Alexa Fluor 546 (1:1000, 1 h; Thermo Fisher Scientific Inc. MA, USA) and Alexa Fluor 488 (1:1000, 1 h; Thermo Fisher Scientific Inc.). In contrast, in the experiment of Fig. (performed *in situ* hybridization and immunostaining simultaneously), NFH antibody and Alexa Fluor 488 were used at the same concentration. After washing with PBS, sections were incubated with DAPI for nuclear staining.

After washing with PBS, sections were encapsulated using fluoromount (Diagnostic BioSystems). We evaluated sections from the cervical and lumbar cord, mainly focusing on the anterior horn and the white matter. The posterior horn was also evaluated.

### Data analysis

**Figure 1:**
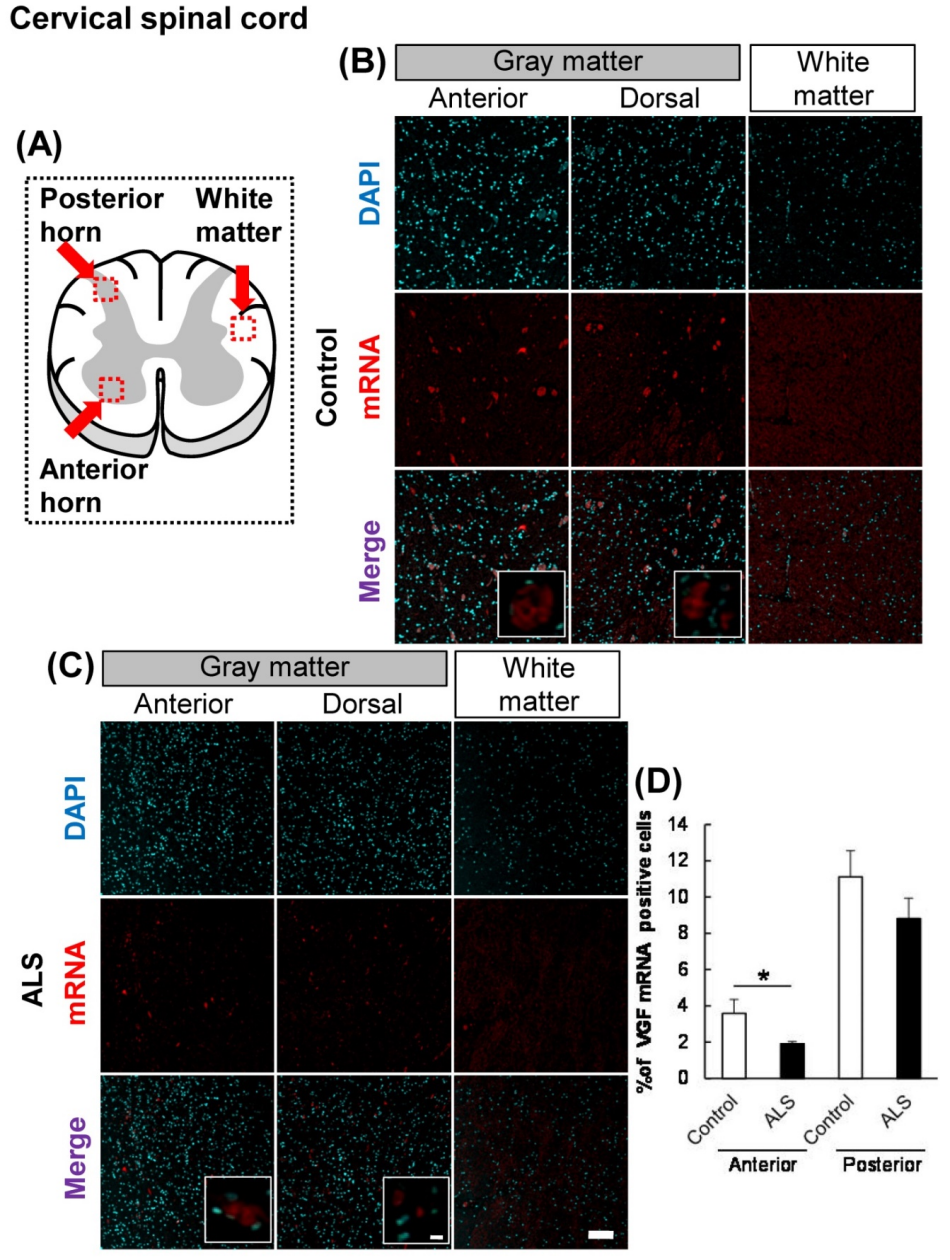
** The localization of *VGF* mRNA in the human cervical spinal cord.** (A) The illustration is a sectional view of the spinal cord. Red squares show the analyzed regions. Anterior horn of the gray matter and the white matter were mainly evaluated. (B, C) Representative images of *in situ* hybridization using RNA probe of human VGF. (B) The images represent the results of control patients and (C) the images represent the results of sporadic ALS patients for gray matter of the anterior horn and white matter in the cervical spinal cord. Scale bars in normal and high magnified images represent 100 or 10 µm, respectively. (D) VGF-positive neuron in anterior and posterior horns, which localized in motor neuron and sensor neuron, respectively, were quantified. VGF-positive cells were decreased in anterior horn of ALS patients as compared with those of control subjects, but not in posterior horn of ALS patients.

**Figure 2:**
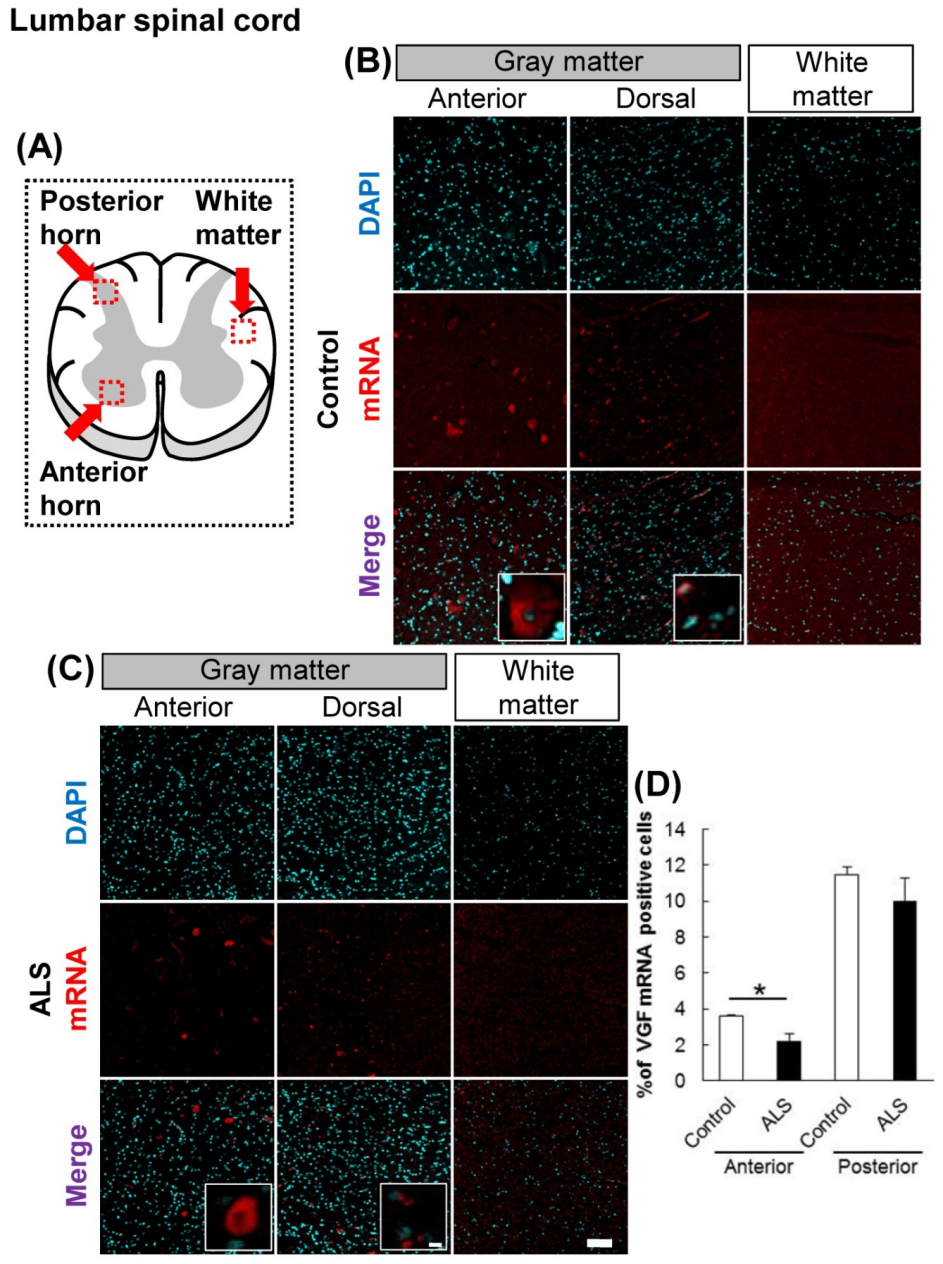
** The localization of *VGF* mRNA in the human lumbar spinal cord.** (A) The illustration is a sectional view of the spinal cord. Red squares show the analyzed regions. Anterior horn of the gray matter and the white matter were mainly evaluated. (B, C) Representative images of *in situ* hybridization using RNA probe of human VGF. The images (B) represent the results of control patients and (C) the images represent the results of sporadic ALS patients for gray matter of the anterior horn and white matter in the lumbar spinal cord. Scale bars in normal and high magnified images represent 100 or 10 µm, respectively. (D) VGF-positive neuron in anterior and posterior horns, which localized in motor neuron and sensor neuron, respectively, were quantified. VGF-positive cells were decreased in anterior horn of ALS patients as compared with those of control subjects, but not in posterior horn of ALS patients.

Images were taken by using a laser confocal microscope (FV10i; Olympus). In figures 1 and 2, images were taken at 20 times magnification. In a blind manner, total cell number (nuclear staining) and VGF mRNA signals were counted using the Image J software (http://rsbweb.nih.gov/ij/). In figure 4, three images were taken in each patient at 40 times magnification. Percentage of *VGF* mRNA positive cell which merged with NFH was calculated.

### Statistical analysis

Data are shown as mean ± S.E.M. The statistical significance of the data was evaluated using Student's *t*-test following a one-way analysis of variance (ANOVA) (JSTAT for Windows; Vector) with *p* < 0.05 indicating a significant difference.

## Results

### *VGF* mRNA localized in the dorsal and anterior horns

First, we evaluated the localization of VGF in the cervical and lumbar spinal cords using *in situ* hybridization. In controls, *VGF* mRNA was localized in both the anterior and posterior horns, and there was no signal in the white matter (Figs. 1, 2). These results suggest that sensory and motor neurons supply VGF to the spinal cord. Moreover, we investigated the expression level of *VGF* mRNA in the spinal cords of ALS patients. In ALS patients, the expression level of *VGF* mRNA significantly decrease in the anterior horn, whereas in the dorsal horn, the expression level of *VGF* mRNA was not changed (Figs. 1, 2).

**Figure 3:**
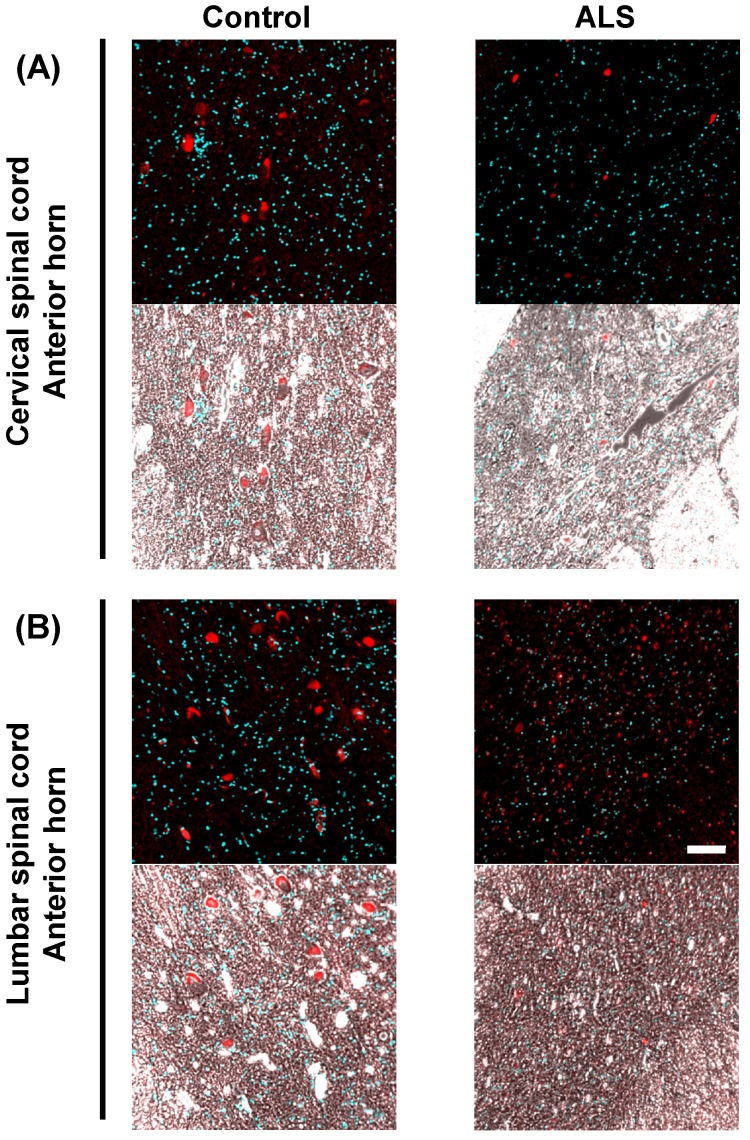
** The localization of *VGF* mRNA in the human spinal cord.** Representative images of *in situ* hybridization using RNA probe of human VGF in the gray matter of the anterior horn. The upper images represent the results of the cervical spinal cord and the lower images represent the results of the lumbar cord. In this figure, we show overlaid fluorescence and phase contrast images to observe the localization of *VGF* mRNA in detail. Scale bar = 100 µm. In the anterior horn of the cervical and lumber cords, VGF mRNA was localized in a motor neuron-like structure.

Subsequently, we observed the localization of *VGF* mRNA in detail by overlaying phase-contrast images on magnified images. In the anterior horn of the cervical and lumber cords, *VGF* mRNA was localized in a motor neuron-like structure (Fig. 3).

### *VGF* mRNA co-localized in motor neuron marker

**Figure 4:**
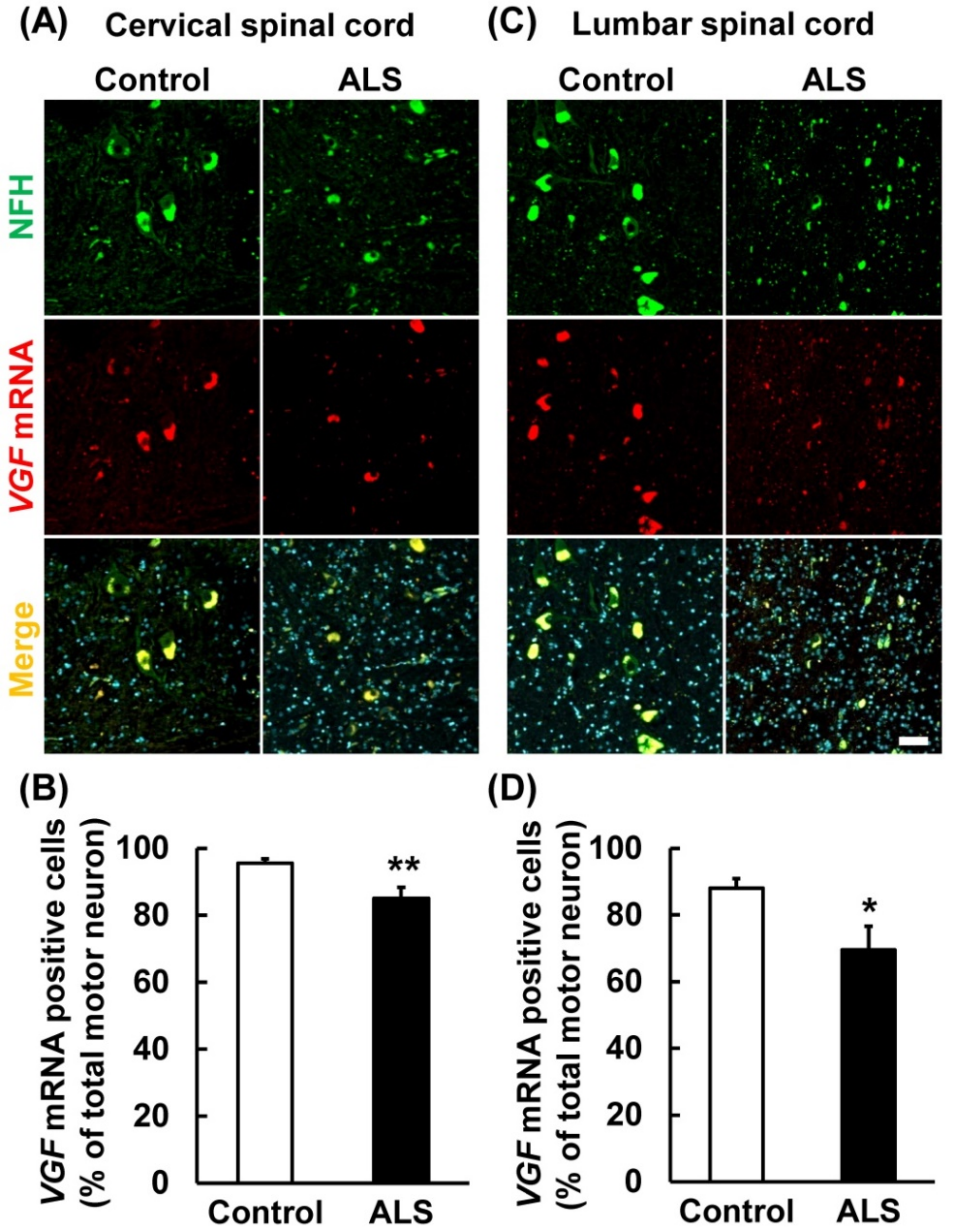
***VGF* mRNA was localized in motor neurons.** The upper images represent the results of *in situ* hybridization and immunohistochemistry in the cervical and lumber spinal cord. The lower graphs represent VGF-positive cell number of NFH-positive motor neurons. Values are given as mean ± S.E.M. (n = 9). *p < 0.05, **p < 0.01 versus control group (Student *t*-test). Scale bar = 50 µm. VGF mRNA positive motor neurons were significantly decreased in both the cervical and lumber spinal cords of ALS patients.

Present results suggest that the *VGF* mRNA is supplied by motor neurons in the anterior horn. Therefore, we carried out immunostaining and *in situ* hybridization simultaneously using a motor neuron marker and VGF RNA probe. *VGF* mRNA was co-localized with NFH (Fig. 4A, C). This result suggests that VGF is produced by motor neurons in the anterior horn. Furthermore, we investigated whether the expression level of *VGF* mRNA is changed in ALS patients. *VGF* mRNA positive motor neurons were significantly decreased in both the cervical and lumber spinal cords of ALS patients (Fig. 4B, D).

### VGF protein localized in motor neurons and decreased in spinal cords of ALS patients

**Figure 5:**
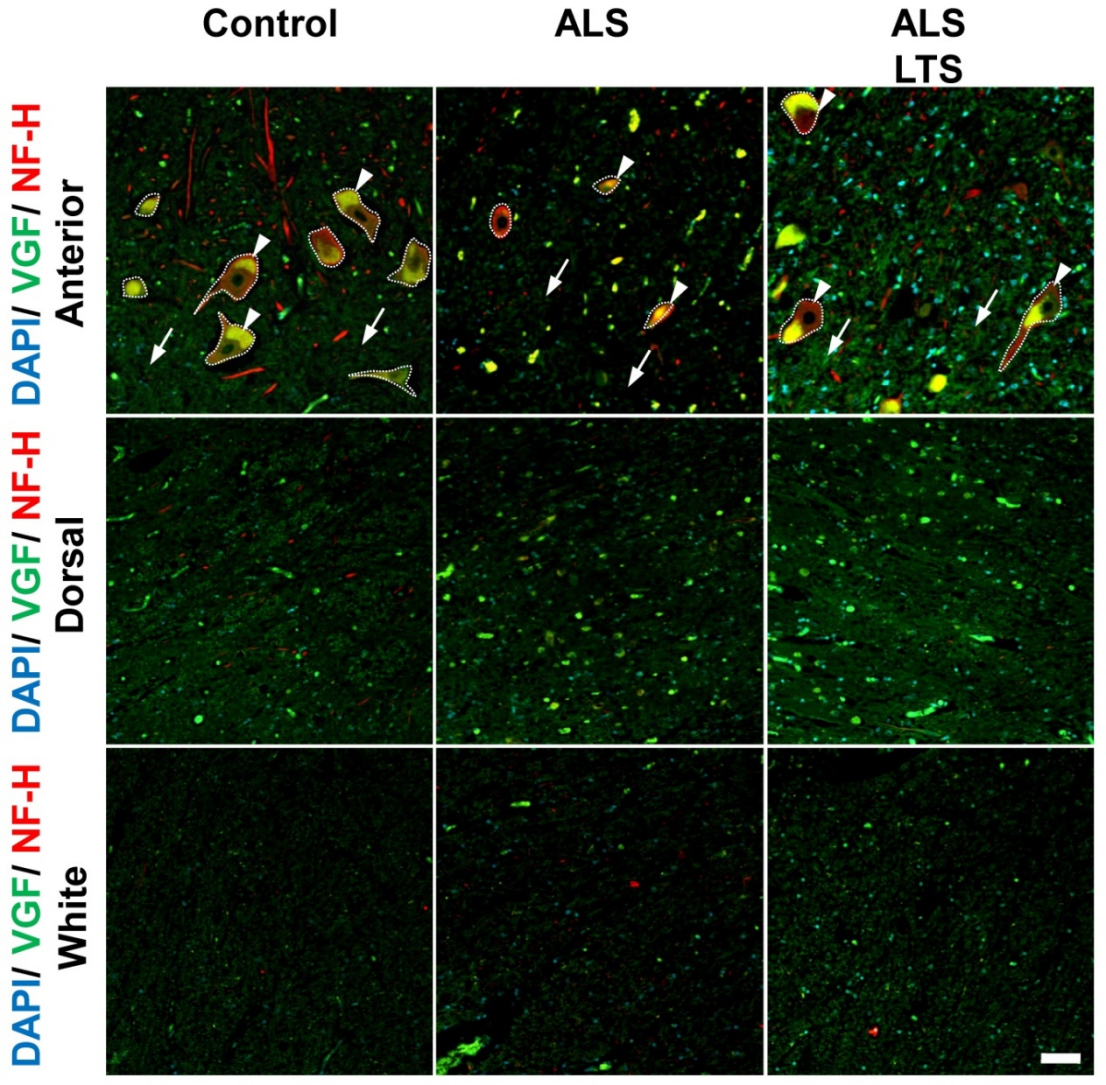
** VGF protein localized in motor neurons and decreased in spinal cords of ALS patients.** The images represent the results of double immunostaining using VGF and NFH antibodies. We observed VGF expression in the anterior and dorsal horns in gray matter and white matter using controls, sporadic ALS patients, and long-term survival sporadic ALS patients. Scale bar = 50 µm. Arrow and arrow head represent intracellular and extracellular areas of motor neurons, respectively. The dotted line represents the profile line of motor neurons. In the ALS patients' motor neurons, the expression level of VGF protein was decreased in both intracellular (arrow head) and extracellular (arrow) areas, while in the dorsal horn and white matter, the expression level of VGF protein was not changed. The immunoreactivity of VGF was maintained in the extracellular region in the long-term survival ALS patients. Moreover, the expression of VGF protein was not different in the white matter or dorsal horn between the control and ALS patients.

To confirm whether the expression of VGF protein is correlated with mRNA expression, we performed double immunostaining using VGF and NFH antibodies. In the control patients, VGF protein was localized in NFH-positive motor neurons and in the dorsal horns; however, there are no signals of VGF in the white matter (Fig. 5). In the ALS patients' motor neurons, the expression level of VGF was decreased in both intracellular (*arrow head*) and extracellular (*arrow*) areas, while in the dorsal horn and white matter, the expression level of VGF was not changed (Fig. 5). These results agree with the localization of *VGF* mRNA. Moreover, we evaluated the expression level of VGF protein in 3 ALS patients with long-term survival 18. The immunoreactivity of VGF was maintained in the extracellular region in the long-term survival ALS patients. On the other hand, the expression of VGF protein was not different in the white matter or dorsal horn between the control and ALS patients (Fig. 5).

## Discussion

In this study, we revealed that *VGF* mRNA and its protein were localized in the dorsal and motor neurons of the anterior horn, while there were no signals in the white matter. Furthermore, VGF-positive motor neurons and extracellular VGF protein were decreased in the spinal anterior horns of ALS patients.

There are some reports that suggest involvement between ALS and VGF expression. The expression level of VGF is decreased in the cerebrospinal fluid (CSF), spinal cords, plasma, and fibroblasts of ALS patients and animal models 12-15. Furthermore, VGF overexpression suppressed cell death in the primary cultured motor neurons obtained from SOD1^G93A^ ALS model mice 19. As for the VGF plasma levels in the patients with ALS, the TLQP peptides, one of the VGF peptides, were decreased in the early phase ALS patients, while VGF C-terminus peptides were decreased in the late phase ALS patients. These data suggests that more detailed studies are needed to determine the involvements of VGF expression in the progression of ALS pathology, and then investigating the localization and expression changes of *VGF* mRNA would provide a new insight into VGF and ALS relationship. Because of these results, we suggest that VGF and its derived peptides could be used as biomarkers for ALS diagnosis. ALS is determined by exclusion of diagnosis from other similar disorders and, therefore, investigation of new specific biomarker is important for treatment of ALS. It was reported that plasma VGF level is decreased in ALS patients 15. However, further investigation will be needed to use VGF as a biomarker for ALS diagnosis.

In the spinal cord, it has been reported that VGF localized in sensory neurons and the expression level of VGF is changed when sensory neuron injury occurs 20,21. In this study, we revealed that *VGF* mRNA was expressed in the dorsal horn, and the expression level of VGF was not changed in ALS patients (Figs. 1, 2). This result indicates that a decrease of VGF expression is not responsible for sensory neurons under ALS pathology. There were few reports that evaluated the details of VGF expression; however, it was already known that VGF immunoreactivity is reduced in the spinal anterior horn of ALS patients and model animals 19. It was previously confirmed that full length VGF and its derived peptide NAPP-129 (VGF_485-615_) are detected in the mouse cervical spinal cord 14, which indicates that VGF is produced in the spinal cord. In this study, we demonstrated that VGF is produced by motor neurons and VGF immunoreactivity was decreased around the motor neurons in ALS patients (Figs. 4, 5). These findings indicate that VGF is mainly provided by motor neurons in the anterior horn.

VGF and its derived peptide have neuroprotective effects in several pathological models such as Huntington's disease 17. These results indicate that the decrease of VGF expression in ALS patients is caused by impaired production of motor neurons, and this promotes the disease progression. Therefore, VGF and its derived peptide may be used as therapeutic targets as well as a biomarker of ALS. In this study, we evaluated the number of *VGF* mRNA positive motor neurons and these were decreased in ALS patients (Fig. 4). However, the decrease was moderate and VGF protein-positive motor neurons were maintained while the immunoreactivity of VGF remarkably decreased in the extracellular region (Fig. 5). In contrast, the immunoreactivity of VGF was maintained in the extracellular region in the long-term survival ALS patients (Fig. 5). In the present study, as only 3 patients are analyzed for long-term survival ALS patients, large scale studies are needed. There are few reports which proteins directly interact with VGF such as binding to C3a receptor and regulate Ca^2+^ influx 22, however, it was unknown that the most of VGF derived peptides interact directly. Especially, the interacting partner of AQEE-30 which has neuroprotective effects and neurite outgrowth remains to be elucidated. It will be helpful for understanding of ALS pathology by elucidating interacting partner of VGF. Furthermore, VGF has a function that supports the formation of a vesicle and interacts with chromogranin B, which regulates secretion of proteins and neurotransmitters 23 and its expression level is changed in ALS patients 24. Furthermore, it was reported that the VGF decrease in the CSF of ALS model mice occurred before muscle weakness, which was a prominent symptom of the pathological condition of ALS 13. These findings indicate that the decrease of VGF in the CSF of ALS patients occurs by inadequate secretion of VGF. Furthermore, there are some reports that exogenously suggested that VGF peptides such as AQEE-30 exerted neuroprotective effects against stress conditions 17. Zhao et al. 13 have also reported that VGF is markedly decreased in the spinal cords of the familial ALS mice with mutated SOD1^G93A^ compared with that in their WT littermates, and that exogenous VGF expression by adenoviral mouse VGF transfection in the primary spinal cord neurons from SOD1^G93A^ mice can protect neurons against excitatory amino acid-mediated injury. Therefore, it is indicated that secreted VGF is more important to cell survival and inadequate secretion of VGF may be involved in the progression of ALS pathology.

In conclusion, we revealed that VGF is produced by motor neuron in human spinal cord and its expression level was decreased in ALS patient and these results suggest VGF may be a therapeutic target of ALS.

## Figures and Tables

**Table 1:** Spinal cords from patients with sporadic ALS or other diseases

Characteristic	Control	ALS patient	Long term survival ALS patient
**Patients (n)**	9	9	3
**Male (n)**	6	8	3
**Female (n)**	3	1	0
**Age (y)**	69.8 ± 4.6	70.9 ± 2.7	76.3 ± 2.3
Postmortem delay*(h) (average)	2 ~ 5 (2.8 ± 0.32)	1.5 ~ 14 (3.9 ± 1.4)	7.0 (7.0 ± 3.6)
**Storage time (y)**	8.3 ± 1.5	11.1 ± 1.5	19.4 ± 4.4

Age data are presented as mean ± S.E.M. Spinal cord samples were acquired from nine patients with sporadic ALS, three ALS patients with long-term survival, and nine patients with other diseases. Formalin-fixed samples were stored at room temperature until sectioning. *, There was one ALS patient who have no data about postmortem delay. Storage time means the time from the date of death to slice into sections. n, number; h, hours; y, years.
